# Sciatica-like pain caused by cervical spondylotic myelopathy: four case reports and systematic review

**DOI:** 10.3389/fmed.2024.1429618

**Published:** 2024-08-01

**Authors:** Changsheng Han, Jingming Wang, Lei Wang, Qinglei Gong, Weimin Huang

**Affiliations:** ^1^Orthopedic Department, 960 Hospital of People’s Liberation Army, Jinan, Shandong, China; ^2^Radiology Department, 960 Hospital of People’s Liberation Army, Jinan, Shandong, China

**Keywords:** sciatica, cervical spondylotic myelopathy, misdiagnose, case report, systematic review

## Abstract

**Objective:**

This study aimed to demonstrate and characterize a rare cervical spondylotic myelopathy (CSM) with sciatica-like pain as the main clinical manifestation through case presentation and systematic review.

**Methods:**

Four cases of CSM with sciatica-like pain as the main clinical manifestation were retrospectively studied. A systematic review of electronic databases such as PubMed, Embase, and Web of Science was conducted to explore the clinical characteristics of CSM with sciatica.

**Results:**

All four cases of CSM symptomatic of sciatica-like pain were initially diagnosed with lumbar degenerative conditions. However, due to the presence of neurological signs such as hyperactive tendon reflexes, a positive Babinski sign, or a Hoffmann sign, they underwent further cervical MRI scans. Eventually, all four cases were diagnosed with CSM and experienced relief from sciatica after cervical decompression surgery. The systematic review analyzed a total of four studies with a combined sample size of 10 cases, all of whom experienced a reduction in sciatica-like pain following cervical decompression surgery.

**Conclusion:**

CSM symptomized by sciatica-like pain can often be misdiagnosed as lumbar degenerative disease. Preoperative abnormal neurological signs associated with CSM may aid in diagnosing this condition. In addition, the clinical presentation of hyperextension of the cervical spine resulting in worsening lower limb pain may serve as diagnostic indicators for this disease.

## Introduction

Cervical spondylotic myelopathy (CSM) is a prevalent condition, with a reported lowest incidence of 1.6 individuals per 100,000 population (1). This condition primarily occurs due to the degeneration and protrusion of intervertebral disks, as well as hypertrophy of the ligamentum flavum, resulting in compression of the cervical spinal cord (2). Common clinical manifestations include neck or shoulder pain, limb numbness, and abnormal gait (3, 4). Diagnosis of typical CSM can be made based on symptoms, physical examination, magnetic resonance imaging (MRI), and neurophysiologic outcome findings (5–7).

Previous studies have found that CSM can present symptoms similar to those of other neurospinal myelopathies (8). Cases of CSM with atypical manifestations have been reported, including knee joint pain (9), low back pain (10), hemiplegia (11), and mechanical neck pain (12). The presence of these atypical symptoms adds to the difficulty of diagnosing CSM.

Among the various atypical clinical presentations of CSM, it is crucial to give more attention to sciatica-like pain. CSM can cause spinal cord parenchymal injury and affect spinal cord blood supply, which may potentially lead to damage of spinal cord cells and neural networks and ultimately result in dysfunction of spinal cord functions. This may manifest as nerve damage affecting the lower limbs and symptoms of sciatica. Due to the complexity of spinal cord function, the clinical manifestations are also complex and diverse, and its pathological mechanism is not completely clear, which needs further research (13, 14). In the clinic, this particular clinical manifestation is often disregarded and misdiagnosed, especially considering that many patients with CSM also have lumbar degenerative diseases. In some instances, patients even undergo unnecessary lumbar spine surgery. Although previous studies have reported similar cases, the number of documented cases has been limited. This study contributes by reporting four cases of sciatica as a clinical manifestation of cervical spondylosis observed at our center. The aim is to provide a detailed description of this unique clinical condition through case reports and a systematic review.

## Case presentation

### Case 1

**Figure 1:**
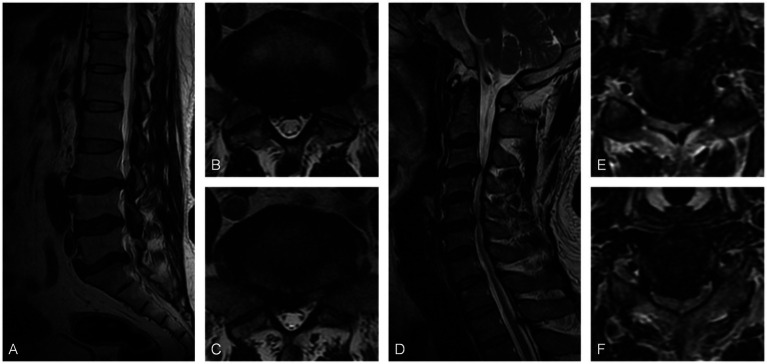
**(A)** Sagittal MRI scan and **(B,C)** axial MRI scans showed the L5/S1 disk protruding to the central right, leading to compression of the nerve root. **(D)** Sagittal MRI scan and **(E,F)** axial MRI scans showed C3/4 cervical disk protrusion to the left posterior and C5/6 disk protrusion to the right of the central posterior. These protrusions caused compression of the spinal cord and resulted in abnormal signals within the spinal cord.

A 51-year-old man with a complaint of left lower limb pain for 2 years was admitted to our hospital. The pain radiated from the buttocks down the lower limbs to the feet, with a visual analog score (VAS) score of 7. The patient had been treated conservatively with oral analgesics. The initial physical examination revealed a reduced skin sensation in the left foot. Electromyography did not show any characteristic changes. MRI scans demonstrated intervertebral disk protrusion to the right of the center at the L5/S1 level. The initial diagnosis was lumbar disk herniation. However, a further physical examination revealed a positive bilateral Hoffmann sign. Subsequently, a cervical spine MRI was performed and revealed that cervical disk herniation compresses the spinal cord and causes abnormal signals in the spinal cord at the C3/4 and C5/6 levels. Finally, the patient was diagnosed with CSM. A posterior cervical laminoplasty (C3–C6) was performed, and left lower limb pain was significantly relieved postoperatively with a VAS score of 2. During the 3 years of postoperative follow-up, the patient had complete relief of symptoms and did not report any complications (Figure 1).

### Case 2

**Figure 2:**
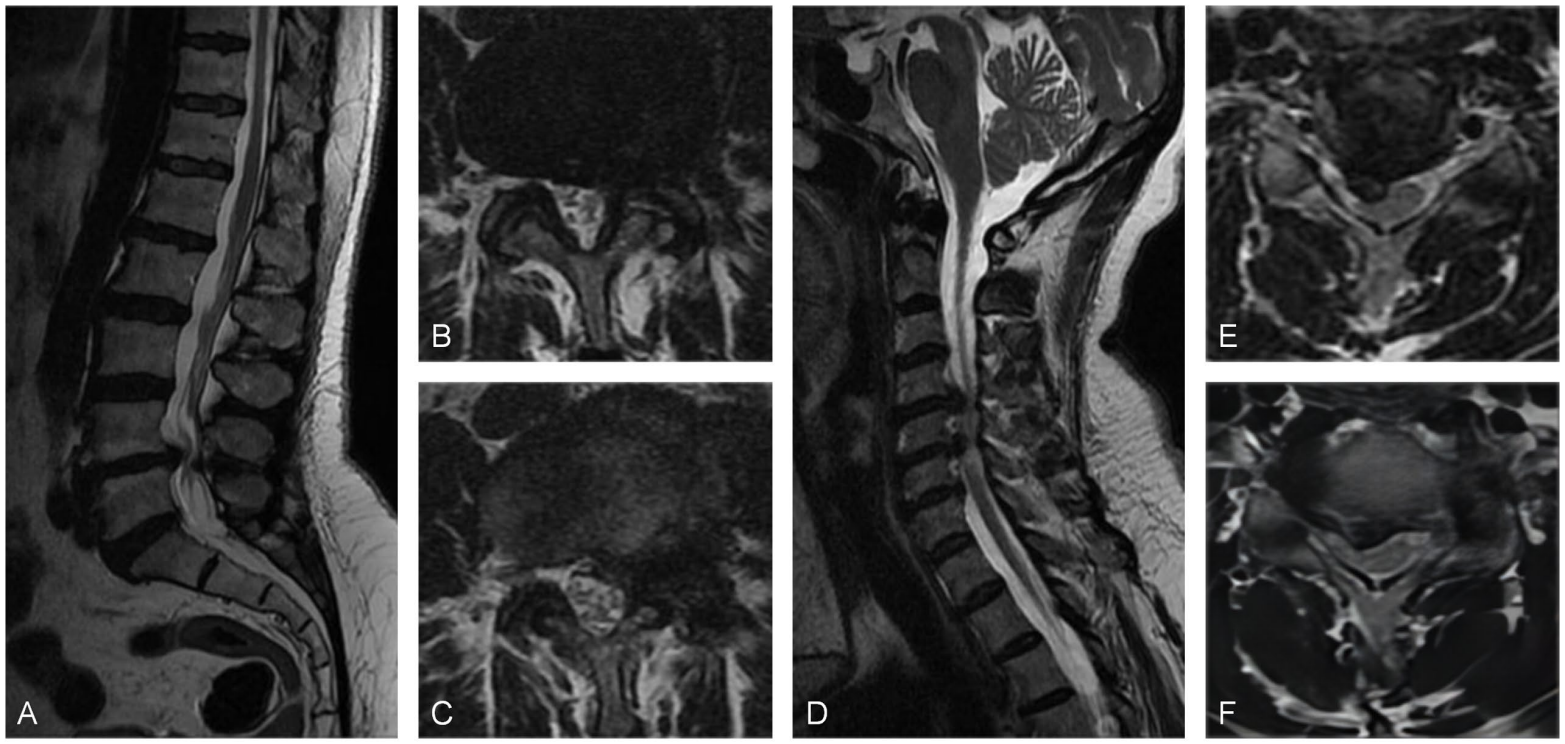
**(A)** Sagittal MRI scan and **(B,C)** axial MRI scans revealed a posterior protrusion of the L4/5 disk, causing spinal canal stenosis. **(D)** Sagittal MRI scan and **(E,F)** axial MRI scans showed disk protrusion at C4/5 and C5/6, resulting in the compression of the spinal cord.

A 53-year-old woman with a 2-year history of sciatica and 3 months of claudication with a VAS score of 7 was admitted to the hospital. She used to receive physical therapy such as acupuncture and massage at a local hospital. Physical examination showed tibialis anterior muscle strength was grade IV, bilateral knee reflexes were slightly active, and lower limb pain was aggravated by cervical hyperextension. Electromyography did not show any abnormal changes. MRI scans showed a protrusion of the L4/5 intervertebral disk to the left and spinal canal stenosis at the L4/5 level. The preliminary diagnosis was lumbar disk herniation with contralateral sciatica. However, following a right L5 nerve root block, the patient did not experience any relief. A thorough neurological examination revealed a suspiciously positive bilateral ankle clonus. Subsequent cervical spine MRI examinations revealed herniation of the C4/5 and C5/6 intervertebral disks to the right, leading to spinal cord compression. Posterior cervical laminoplasty (C3–C7) was performed, and the patient experienced significant relief immediately postoperatively. During the 17-month follow-up period, the sciatica-like pain completely resolved (Figure 2).

### Case 3

**Figure 3:**
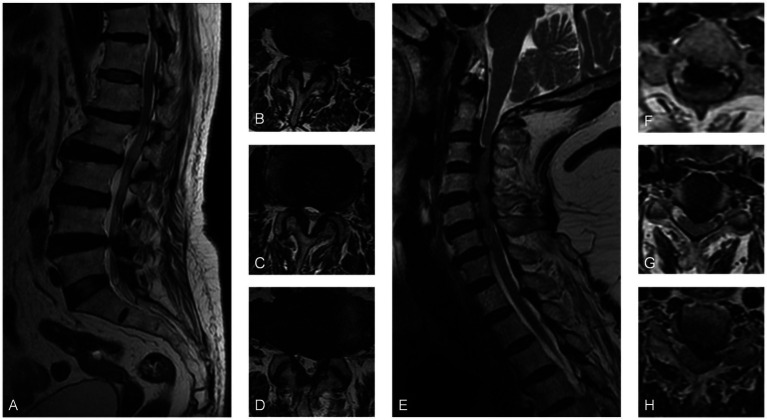
**(A)** Sagittal MRI and **(B–D)** axial MRI revealed L4/5 disk protrusion and spinal canal stenosis. **(E)** The sagittal MRI scan and **(F–H)** the axial MRI scans demonstrated multi-segmental disk herniation from C2 to C7, hypertrophy of the ligamentum flavum causing cervical spinal canal stenosis, and spinal cord compression at the corresponding levels.

A 71-year-old woman was admitted to the hospital due to sciatica that had been present for 6 years and worsened for 2 weeks. The pain radiated from the right hip to the foot and worsened when standing with a VAS score of 6. She was usually treated with topical analgesic ointment. Physical examination showed positive Lasegue and Bragard signs in the left leg. MRI scans showed that the left spinal canal stenosis was caused by a herniated disk at L4/5 and hypertrophy of the ligamentum flavum. Electromyography showed no obvious abnormalities. The initial diagnosis was lumbar spinal stenosis. However, due to the inconsistency of the symptom side and the radiographic protruded side, a further physical examination was performed, which revealed that bilateral Babinski signs were suspiciously positive. Then a cervical spine MRI scan was performed and revealed spinal cord compression caused by multi-segment intervertebral disk herniation and hypertrophy of the ligamentum flavum. Following the posterior cervical laminoplasty procedure (C3–C7), the patient experienced significant alleviation of pain in the right lower limb, as evidenced by a decrease in the VAS score to 1 postoperatively. Throughout the 21-month follow-up period, the patient did not report any further complaints related to lower extremity pain (Figure 3).

### Case 4

**Figure 4:**
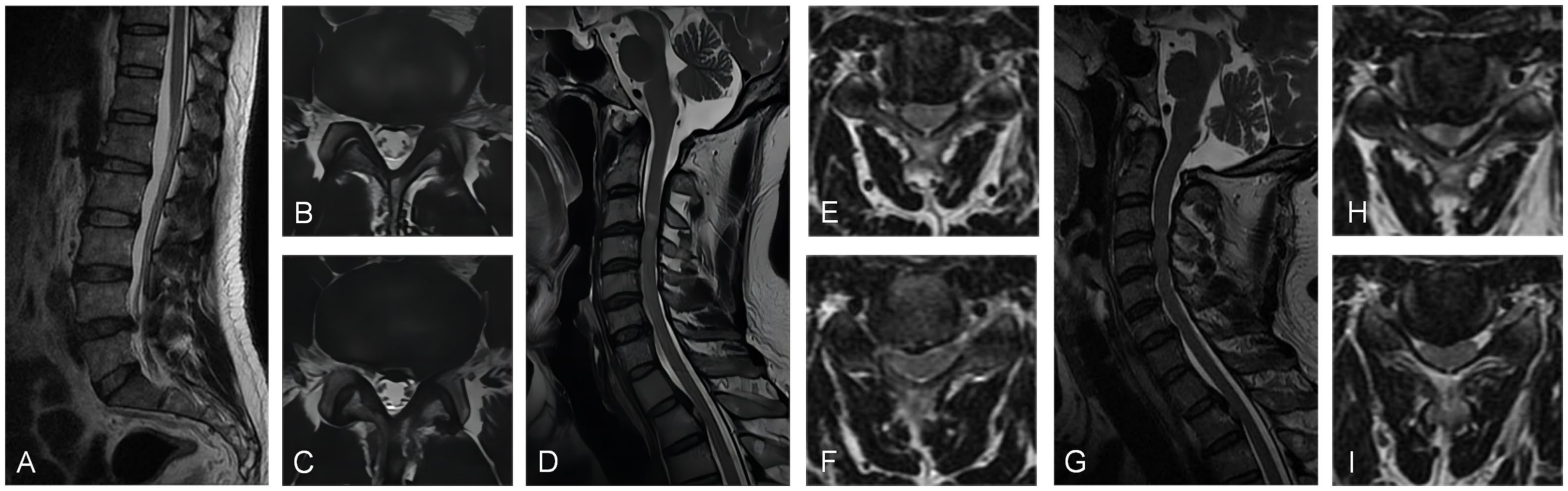
**(A)** Sagittal MRI scan and **(B,C)** axial MRI scans exhibited posterior protrusion of the L4/5 disk to the central left. **(D)** The sagittal MRI scan and **(E,F)** axial MRI scans demonstrated posterior protrusion of the C3/4 and C4/5 cervical disk, resulting in mild spinal cord compression with abnormal signal. **(G)** The sagittal MRI scan and **(H,I)** axial MRI scans in the hyperextended position demonstrated that the C3/4 and C4/5 cervical disks had significant compression of the spinal cord.

A 47-year-old man was admitted to the hospital with a diagnosis of lumbar disk herniation due to sciatica for 2 months. The preoperative VAS score was 6. The patient had been taking oral analgesics for 2 months to relieve pain, but their effectiveness had been deteriorating. Physical examination revealed decreased skin sensation on the right side of the lower extremity, a positive Babinski sign on the left side, and increased pain in the lower extremity during cervical hyperextension. A lumbar MRI scan showed a central protrusion of the L4/5 vertebral body. Electromyography did not detect any obvious abnormalities. Further cervical spine MRI examination revealed multi-segment intervertebral disk herniation, mild spinal cord compression, and an abnormally high signal in the C3/4 spinal cord. Since the cervical spine MRI could not fully explain the high signal intensity in the spinal cord and the lower limb symptoms, a further cervical spine hyperextension MRI was performed. The results confirmed obvious compression of the C3/4 segment of the spinal cord by the herniated intervertebral disk. The patient underwent anterior cervical discectomy and fusion (ACDF), which significantly relieved the sciatica-like pain in the right lower limb postoperatively. During the 11-month postoperative follow-up, the patient experienced only slight numbness in the lower limbs. The VAS score at the last follow-up was 1 point (Figure 4).

## Systematic review

### Literature search

Electronic databases including PubMed, Embase, and Web of Science were searched to identify previously reported cases of CSM with sciatica-like pain. The language of the publications was limited to English. The published time was not restricted. The search terms were (“cervical spondylotic myelopathy” OR “cervical disc herniation”) AND (“unilateral lower limb pain” OR “sciatica” OR “tract pain” OR “funicular pain” OR “radiating pain to lower”) by title/abstract and/or topic. In addition, a supplementary search of references cited in all relevant articles was performed. Studies that did not provide sufficient information for further analysis were excluded.

### Data extraction

Data extracted from eligible papers included baseline characteristics, neurological examinations, imaging findings, and clinical outcomes. Data collection was conducted independently by two reviewers using a standard form. If disagreements persisted, arbitration was conducted by a third review author.

### Quality assessment

The Joanna Briggs Institute (JBI) Case Reports Quality Assessment Tool was used to assess the risk of bias in the included literature. The quality assessment tool includes eight points to evaluate the quality of case reports from the aspects of patient history, clinical manifestations, diagnosis, treatment, etc. Each item was judged by “yes,” “no,” “unclear,” and “not applicable,” and the results were cross-checked by two researchers independently. We determined that articles achieved the adequate standard of quality required for their inclusion if three-quarters or more of the questions were answered “Yes” (Table 1).

**Table 1:** Study quality by the Joanna Briggs Institute (JBI) case reports quality assessment tool.

Author	1	2	3	4	5	6	7	8	Quality Assessment
Langfitt (15)	Yes	Yes	Yes	Yes	Yes	Yes	Yes	Yes	High Quality
Yoon (16)	Yes	Unclear	Yes	Yes	Yes	Yes	No	Yes	High Quality
Kozaki (17)	Yes	Yes	Yes	Yes	Yes	Yes	Yes	Yes	High Quality
Crocombe (18)	Yes	Yes	Yes	Yes	Yes	Yes	No	Yes	High Quality
Chan (19)	Yes	Yes	Yes	Yes	Yes	Yes	No	Yes	High Quality
Gao (20)	Yes	Yes	Yes	Yes	Yes	Yes	No	Yes	High Quality

### Literature selection

A total of 151 documents were retrieved. Following deduplication exclusion, exclusion by titles and abstracts, and exclusion by full texts, a total of five documents with 10 cases were included in this study. All the studies achieved an adequate level of quality to warrant their inclusion.

### Data analysis

The baseline characteristics of the included studies are shown in Table 2. There were three women and seven men, with a mean age of 53.4 years old (range 34–75). Five patients underwent ACDF, two patients underwent dome-like laminectomy, and one patient underwent MOC (multilevel oblique corpectomy) surgery. All patients experienced sciatica relief after cervical spine surgery.

**Table 2:** Characteristics of included cases.

Author	Country	Journal	Published year	Case number	Gender	Age (years)	Side	Symptom duration	Index levels	Electromyography examination	Surgical procedure
Langfitt (15)	USA	JAMA	1967	2	M	47	Bilateral	10 Months	C5-C7	/	Posterior cervical laminectomy
					M	51	Bilateral	1 Years	C3-C6	/	Posterior cervical laminectomy
Yoon (16)	USA	Journal of Clinical Neuroscience	2019	1	M	51	Left	3 Days	C6-C7	/	ACDF
Kozaki (17)	Japan	BMC Musculoskeletal Disorders	2020	1	F	75	Bilateral	6 Months	C3-C7	Neurogenic changes below the C5 level.	Dome-like laminectomy
Crocombe D (18)	USA	British Journal of General Practice	2017	1	M	42	Bilateral	4 Weeks	C6-C7	/	ACDF
Chan (19)	Malaysia	European spine journal	2011	2	F	72	Right	1 Year	C3-C5	/	MOC
					M	61	Right	4 Months	C4-C6	Right L5 radiculopathy	Dome-like laminectomy
Gao (20)	China	Journal of International Medical Research	2023	3	F	40	Left	6 Months	C4-C6	/	ACDF
					M	34	Right	3 Months	C5-C6	/	ACDF
					M	61	Left	5 Years	C4-C6	/	ACDF

Gender: F, female; M, male.

Electromyography examination: missing: /.

Surgical procedure: ACDF, anterior cervical discectomy and fusion; MOC, multilevel oblique corpectomy.

## Discussion

This study presents four cases of CSM with sciatica-like pain. All patients were initially diagnosed with lumbar degenerative diseases. However, due to the atypical imaging findings of the lumbar spine and the presence of abnormal neurological signs, further cervical MRI scans were performed, and the final diagnosis was CSM.

The diagnostic approach for sciatica-like pain caused by CSM remains a topic of debate. Previous studies have shown that myelography, MRI, electromyography, and cervical nerve root block examinations for the diagnosis of CSM can provide some help (21, 22). Scott believed that myelography was an effective way to diagnose a group of conditions in which compression of the spinal cord caused symptoms in the lower extremities and to help relieve pain with surgery (23). However, myelography is a highly invasive procedure, and adverse reactions to the agent are common, such as nausea, headache, rash (24), and even more severe spinal cord injury (25). Electromyography also contributes to the diagnosis of this disease (5). Although it cannot directly assess functional status, it can effectively distinguish the differences between spinal cord lesions and peripheral nerve lesions or nerve root lesions. According to reports, electromyography such as spinal cord evoked potentials, motor withdrawal potentials, and somatosensory evoked potentials can be used to diagnose spinal cord compression with fewer symptoms in the upper limbs (26, 27). Neo et al. argue that the only definitive diagnosis can be made through the relief of symptoms after surgery (28). Chan et al., on the other hand, suggested that cervical nerve root block can effectively diagnose this condition based on two patients who experienced relief from preoperative nerve root block (19). However, it is important to note that cervical nerve root block is an invasive procedure and carries risks such as vascular injury, nerve injury, and spinal cord infarction. Furthermore, some reports question the accuracy of cervical nerve root block (29–32).

Of the four patients reported in the current study, it is noteworthy that all cases had neurological signs associated with CSM. In the context of diagnosing sciatica caused by lumbar degenerative diseases and CSM, neurological signs associated with CSM may provide valuable insights for differential diagnosis. Previous studies have further enhanced this understanding. Crocombe et al. reported a case of back and leg pain resulting from cervical disk prolapse. They observed preoperative hyperreflexia in the patellar tendon and a positive ankle clonus (18). In a similar study, Gao et al. found positive Hoffmann and Babinski signs, as well as ankle clonus, during the preoperative physical examination of patients experiencing lower limb sensory disturbance caused by cervical cord compression (20).

Similar neurological examinations have been previously reported, showing atypical manifestations due to cervical and thoracic spinal cord compression. Secer et al. reported a case series of patients with tract pain caused by CSM, all of whom presented with abnormal physical signs, including a positive Hoffman sign or hyperreflexia in the patellar tendon (33). Cho et al. reported two cases of sciatica-like pain resulting from thoracic disk prolapse. The neurologic examination revealed abnormal findings, such as hyperreflexia patellar tendon, a positive ankle clonus, and a positive Babinski sign (34). Golbakhsh et al. reported neurological abnormalities, including hyperreflexia in the patellar tendon reflex and positive ankle clonus, in a case of back pain caused by thoracic disk herniation (35). Interestingly, lower extremity symptoms were significantly improved in all patients who underwent cervicothoracic spine surgery.

Additionally, we observed an intriguing sign during the physical examination of the patients. In cases 2 and 4, when the neck was hyperextended, there was an exacerbation of lower limb pain symptoms. Although this sign did not appear in cases 1 and 3 and has not been mentioned in previous case reports of sciatica caused by CSM, we believe it may hold diagnostic value for CSM leading to sciatica. However, the specificity and sensitivity of this sign for disease diagnosis still require further verification with more cases.

The pathogenesis of sciatica caused by CSM remains unclear. Sciatica-like pain caused by spinal cord compression is thought to be due to the stimulation of the ascending spinal cord tracts and thalamus in patients with sensory disturbances such as radiating, diffuse, or burning pain (17, 19, 36). The ascending pathways, including the fasciculus cuneatus, spinocerebellar tract, and spinothalamic tract, are responsible for transmitting superficial sensations such as pain, temperature, touch, and pressure, as well as proprioception from the trunk and limbs. The corticospinal tract is the largest and most crucial descending pathway and is responsible for transmitting motor commands that control skeletal muscle, particularly the distal muscles of the limbs. Within the corticospinal tract and the spinothalamic tract, nerve fibers are organized in a topographical manner from the outermost to the innermost regions, corresponding to the sacral, lumbar, thoracic, and cervical fibers. The lumbar and sacral fibers are located laterally and are therefore more susceptible to compression injuries when the spinal cord is subjected to pressure (13). Spinal cord compression can also cause ipsilateral motor weakness due to the downward stimulation of the corticospinal tract. Spinal cord compression may lead to damage to the cortical spinal tract, resulting in manifestations of upper motor neuron damage such as increased muscle tone, enhanced tendon reflex, and pathological signs (37). Therefore, compression of the cervical or thoracic cord is often accompanied by ipsilateral motor weakness and contralateral burning pain (15, 34, 38). Compression of the spinal cord may either stimulate the spinal-thalamic tract, interpreting the stimulation as radiation pain in the lower extremity, or it may relieve the inhibition of the spinal pathway that normally suppresses pain signals (19). This lack of inhibition can be perceived by the brain as pain (34, 36, 39). However, this hypothesis cannot fully explain the observation that not all patients presented with sciatica on the opposite side of the compressed spinal cord in our study and other reports.

An interruption of the spinal cord blood supply should be considered a potential cause of this tract pain (34). The arteries in the anterior and posterior parts of the spinal cord have limited blood flow, with the anterior spinal artery being the main supplier, providing blood to the anterior two-thirds of the spinal cord. The T4 level in the spinal cord is a critical region where the anterior spinal artery divides. When cervical spinal lesions compress the anterior spinal artery, it can lead to ischemia in the thoracic spinal cord near the T4 level, and approximately one-third of patients might experience pain radiating to the lower extremities (20, 40, 41).

This study has several limitations. First, due to the relative rarity of such diseases in clinical practice, the number of cases reported in this study was relatively small. Second, based on the current cases presented in the current study and previous reports, it is not possible to draw any conclusions regarding the presence of specific electromyographic and imaging features for patients with sciatica-like pain caused by CSM. Further multi-center studies with large sample sizes are needed to address these limitations.

## Conclusion

CSM symptomized by sciatica-like pain can often be misdiagnosed as lumbar degenerative disease. Preoperative abnormal neurological signs associated with CSM may aid in diagnosing this condition. In addition, the clinical manifestations of hyperextension of the cervical spine leading to aggravation of lower limb pain are diagnostic clues for this disease.

## Data availability statement

The raw data supporting the conclusions of this article will be made available by the authors, without undue reservation.

## Ethics statement

The studies involving humans were approved by Ethics committee of 960 Hospital of PLA. The studies were conducted in accordance with the local legislation and institutional requirements. Written informed consent for participation in this study was provided by the participants’ legal guardians/next of kin. Written informed consent was obtained from the individual(s) for the publication of any potentially identifiable images or data included in this article.

## Author contributions

CH: Formal analysis, Methodology, Software, Writing – original draft. JW: Conceptualization, Methodology, Software, Supervision, Writing – review & editing. LW: Data curation, Formal analysis, Writing – original draft. QG: Conceptualization, Data curation, Formal analysis, Writing – original draft. WH: Conceptualization, Formal analysis, Funding acquisition, Methodology, Supervision, Visualization, Writing – review & editing.
